# Nasal NK/T cell lymphoma presents with long-term nasal blockage and fever: a rare case report and literature review

**DOI:** 10.18632/oncotarget.7386

**Published:** 2016-02-14

**Authors:** Hai Zou, Ke-Hua Pan, Liang Wu, Hong-Ying Pan, Ya-Hui Ding, Ming-Hua Zheng

**Affiliations:** ^1^ Department of Infection Diseases, Zhejiang Provincial People's Hospital, Hangzhou, China; ^2^ Department of Radiology, The First Affiliated Hospital of Wenzhou Medical University, Wenzhou, China; ^3^ Department of Pathology, The First Affiliated Hospital of Wenzhou Medical University, Wenzhou, China; ^4^ Department of Cardiovascular Medicine, Zhejiang Provincial People's Hospital, Hangzhou, China; ^5^ Department of Infection and Liver Diseases, Liver Research Center, The First Affiliated Hospital of Wenzhou Medical University, Wenzhou, China; ^6^ Institute of Hepatology, Wenzhou Medical University, Wenzhou, China

**Keywords:** NK/T cell lymphoma, fever of unknown origin, clinical manifestation

## Abstract

NK/T cell lymphoma (NKTCL) is a common disease which is a threat to human health. Nasal NKTCL is a rare but serious type of systemic lymphoma because of its high mortality rate and serious complications. In this case report, we describe a male who presented with nasal blockage in the right side, a fever of one month duration and a soy-like, painless and gradually increasing mass in the right submandibular region due to nasal NKTCL. The patient had no significant medical history and the initial clinical symptoms were nasal blockage. Contrast computed tomography showed that the nasopharyngeal mucosa was thickened and that the celiac and retroperitoneal lymphaden was intumescent. Finally a biopsy, guided by nasal endoscopy and examined using flow cytometry confirmed a diagnosis of NKTCL. Nasal NKTCL is rare and has no unique characteristics at first presentation, such as epidemiology and obvious clinical manifestation. As no effective therapy is currently available for this disease, early diagnosis and therapy of nasal NKTCL remains challenging.

## INTRODUCTION

NK/T cell lymphoma (NKTCL) is a common disease which threatens human health. NKTCL is a serious type of systemic lymphoma because of its high mortality rate and serious complications. In this case report, we describe a male who presented with nasal blockage in the right side, a fever of one month duration and a soy-like, painless and gradually increasing mass in the right submandibular region due to NKTCL.

## CASE PRESENTATION

**Figure 1:**
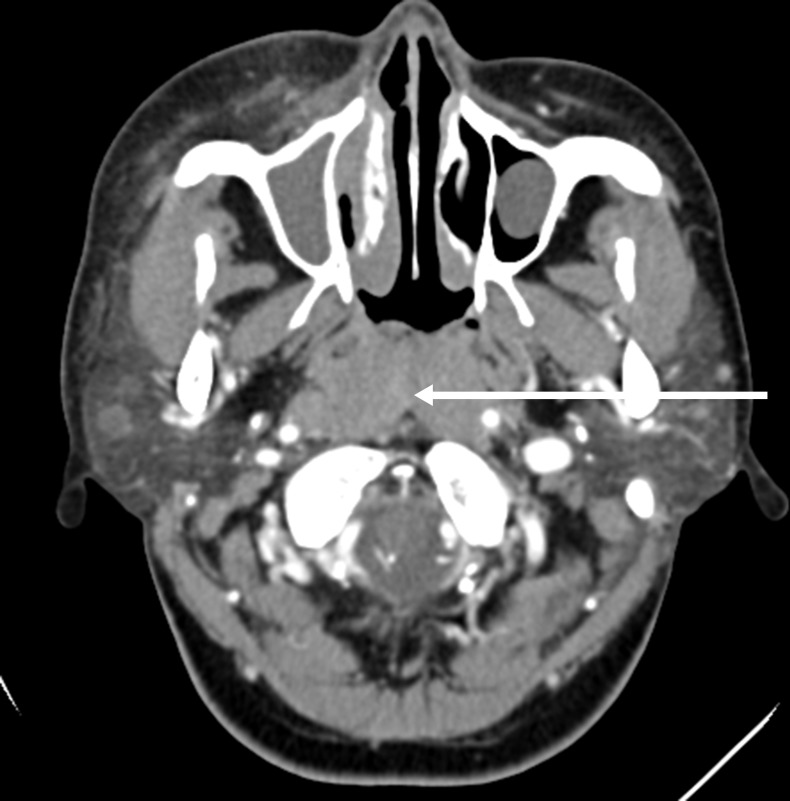
Contrast brain computed tomography shows diffuse swelling of the posterior wall of the pharynx with mild enhancement

**Figure 2:**
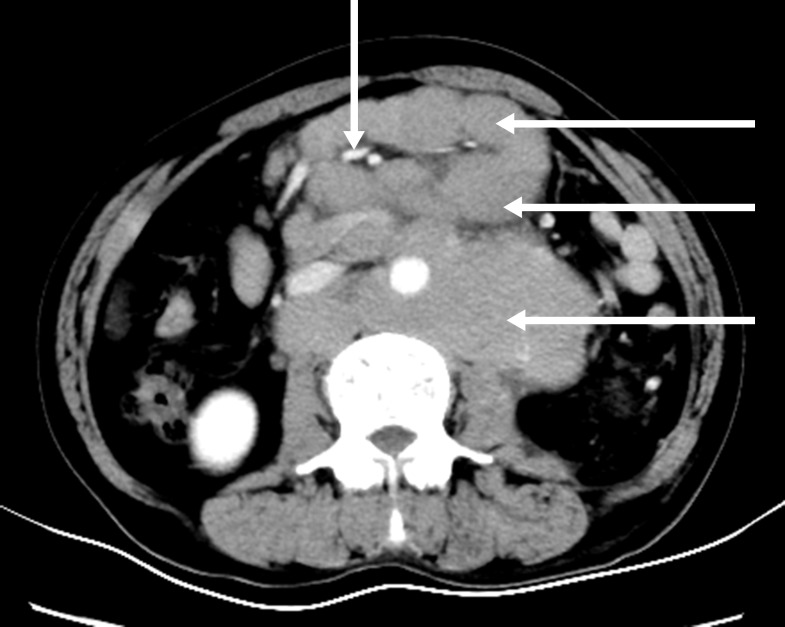
Contrast abdomen computed tomography shows multiple mesenteric and retroperitoneal lymph node enlargement and integration into masses with mild-middle enhancement, the mesenteric vessels embedded (sandwich sign)

**Figure 3:**
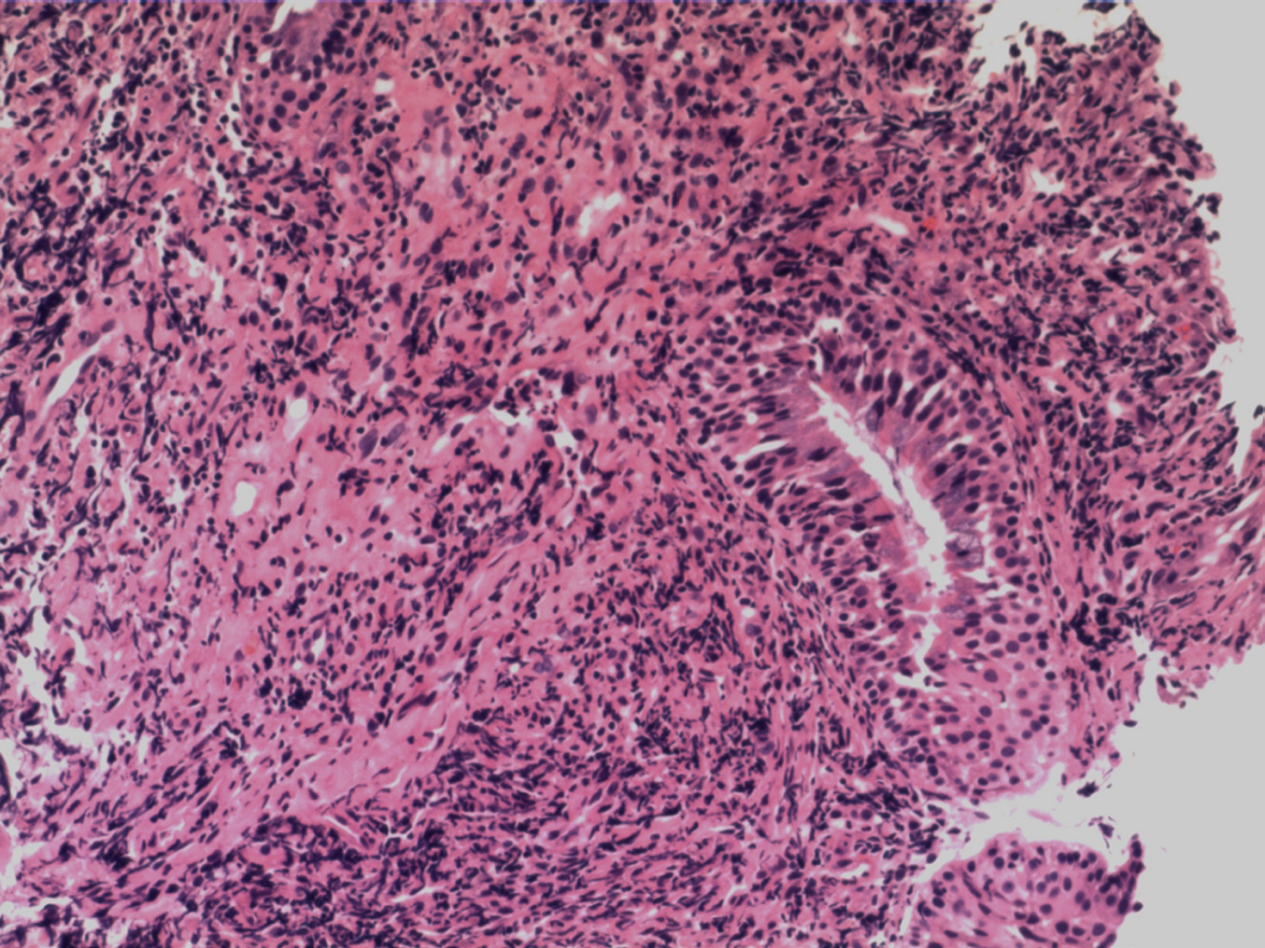
Pathology of the thickened nasopharyngeal mucosa

**Figure 4:**
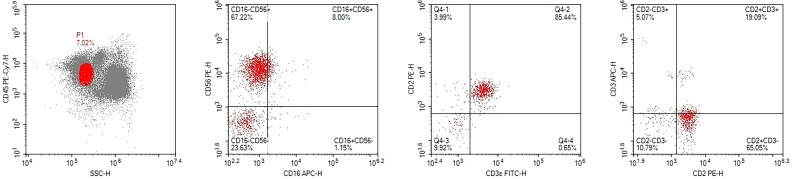
Result of the flow cytometry, based on the biopsy tissue

A 21-year-old male was hospitalized with a ten-month history of nasal blockage and a fever of one month duration. He presented with nasal blockage in the right side at the time of initial presentation, without nasal discharge, headache or tinnitus. Despite symptomatic treatment, he continued to do poorly and had persistent nasal blockage. He developed persistent fever a month ago, beginning at nightfall and peaking to 39°C to 40°C during the night or in the early morning. Meanwhile, a soy-like, painless and gradually increasing mass in the right submandibular area was found which was not responsive to antibiotic treatment. Fever improved when he received dexamethasone, and his temperature increased again when dexamethasone was withdrawn. Contrast computed tomography (CT) showed that the nasopharyngeal mucosa was thickened (Figure 1) and the celiac and retroperitoneal lymphaden was intumescent (Figure 2). Bone marrow aspiration and CT scan of chest and neck show normal. Finally, the biopsy based on nasal endoscopy and examination by flow cytometric methods confirmed a diagnosis of nasal NKTCL (Figure 3, Figure 4). According to the Ann Arbor staging, this patient was IIIB of the disease.

## DISCUSSION

According to the WHO classification in 2001, nasal NKTCL as an independent lymphoma subtype mainly occurring in Asia, Mexico and South America [1], which could have a racial basis, however environmental factors also had to be considered [2].

Our patient first presented with nasal blockage, and the following computed tomography showed that the nasopharyngeal mucosa was thickened. Localized inflammation and tumor are the most likely diagnosis for this person. Nasopharyngitis and nasopharyngeal carcinoma (NPC) could cause thickening of nasopharyngeal mucosa, both of which are associated with viruses. NPC has a high incidence in southern China and Hong Kong, with an incidence of 25 to 50 per 100,000 people [3]. Osteoma, papilloma, and carcinoma are relatively rare but are important potential causes and are responsible for tumors which may cause nasal blockage [4]. The main symptoms are unilateral, slowly increasing blockage of the nose with or without bloody-purulent secretion and impairment of olfaction. In later stages of the disease, epistaxis and facial swelling can occur [5–6] and further progression can lead to ulceration and painful mid-facial destruction. The lymphoma could spread to the skin, gastrointestinal tract, testis, spleen and central nervous system [7]. However, nasal NKTCL could also lead to destruction and loss of function of the upper respiratory tract, especially in the nasal cavity, nasal and paranasal sinuses, and hard palate [8], which has been associated with Epstein barr virus infections [9].

Our patient has a soy-like, painless and gradually increasing mass in the right submandibular area, and has persistent fever beginning at nightfall and peaking during the night or in the early morning. Abdominal primary or metastatic malignant tumors often cause abdominal lymphadenectasis, such as gastric, liver, and colorectal cancer. Mesenteric lymphadenitis can also cause abdominal lymphadenectasis which mainly occurs in children and is usually accompanied with secondary acute upper respiratory tract infection. A CT scan showed mesenteric and retroperitoneal lymphadenectasis. Tuberculous lymphadenitis could occur in the neck, armpits, abdomen and groin. In the early stage, patients commonly present with painless swollen lymph nodes. Some patients experience low-grade fever and night sweats. However, lymphoma is a common cause of lymphadenectasis, of which clinical symptoms are non-specific, although fever is common. Infection, malignancy, collagenous vascular disease could cause fever of unknown origin (FUO). These diseases are usually hidden, and accompanied by atypical symptoms, such as subacute bacterial endocarditis, perforation of the retrocecal appendix, pericolonic abscess, occult liver or splenic abscesses, and prosthetic graft infections, which may easily be missed and eventually present as an undiagnosed prolonged fever [10–12].

However, cancer may account for 20%-30% of fever of FUO, of which lymphoma is the most common cause, accounting for 50%-70% of malignant tumors [13], where the initiating symptom is often lymphadenectasis. It should not be forgotten that because of the increase in the use of polypharmacy, the diagnosis of drug fever as a cause of FUO is relatively more common. Tumor was the most likely diagnosis for this person, but differential diagnoses need to consider inflammatory causes. Patients with long-term use of antibiotics, corticosteroids and immunosuppressants, uncontrolled diabetes mellitus, acquired immune deficiency syndrome are especially susceptible to fungal rhinosinusitis, which have different clinical features, including nasal blockage, runny nose, fever, and maxillary sinus mass. Aspergillus species, or members of the class zygomycetes are the most frequent etiological agents [14–18], which accounts for 80% of nasal symptoms.

Once confirmed as a nasal NKTCL, chemotherapy and radiation combination therapy is considered to be effective. The patient receives a total of 4 cycles of CHOP-L. After achieving complete remission with combined chemo-radiotherapy, hematopoietic stem cell transplantation is also recommended. Meanwhile, immunotherapy with pegylated interferon alpha during chemotherapy and radiation therapy may be also effective. International prognostic index score, near-term complete response rate [19], and other factors could be used to evaluate the prognosis [20].
