# Addendum: Nasal NK/T cell lymphoma presents with long-term nasal blockage and fever: a rare case report and literature review

**DOI:** 10.18632/oncotarget.28285

**Published:** 2023-12-01

**Authors:** Hai Zou, Ke-Hua Pan, Liang Wu, Hong-Ying Pan, Ya-Hui Ding, Ming-Hua Zheng

**Affiliations:** ^1^Department of Infection Diseases, Zhejiang Provincial People’s Hospital, Hangzhou, China; ^2^Department of Radiology, The First Affiliated Hospital of Wenzhou Medical University, Wenzhou, China; ^3^Department of Pathology, The First Affiliated Hospital of Wenzhou Medical University, Wenzhou, China; ^4^Department of Cardiovascular Medicine, Zhejiang Provincial People’s Hospital, Hangzhou, China; ^5^Department of Infection and Liver Diseases, Liver Research Center, The First Affiliated Hospital of Wenzhou Medical University, Wenzhou, China; ^6^Institute of Hepatology, Wenzhou Medical University, Wenzhou, China

**This article has an addendum:** Informed patient consent was obtained for publication of this case report.

Original article: Oncotarget. 2016; 7:9613–9617. 9613-9617. https://doi.org/10.18632/oncotarget.7386

